# Emerging applications of femtosecond laser fabrication in neurobiological research

**DOI:** 10.3389/fchem.2022.1051061

**Published:** 2022-11-04

**Authors:** Mingzhen Tian, Zhuo-Chen Ma, Qingqing Han, Qian Suo, Zhijun Zhang, Bing Han

**Affiliations:** ^1^ Institute of Medical Robotics, Shanghai Jiao Tong University, Shanghai, China; ^2^ School of Biomedical Engineering, Shanghai Jiao Tong University, Shanghai, China; ^3^ Department of Automation, School of Electronic Information and Electrical Engineering, Shanghai Jiao Tong University, Shanghai, China

**Keywords:** femtosecond laser fabrication, micro/nano-robots, 3D scaffolds, drug delivery, two-photon polymerization, micro/nano-structures

## Abstract

As a typical micro/nano processing technique, femtosecond laser fabrication provides the opportunity to achieve delicate microstructures. The outstanding advantages, including nanoscale feature size and 3D architecting, can bridge the gap between the complexity of the central nervous system *in virto* and *in vivo*. Up to now, various types of microstructures made by femtosecond laser are widely used in the field of neurobiological research. In this mini review, we present the recent advancement of femtosecond laser fabrication and its emerging applications in neurobiology. Typical structures are sorted out from nano, submicron to micron scale, including nanoparticles, micro/nano-actuators, and 3D scaffolds. Then, several functional units applied in neurobiological fields are summarized, such as central nervous system drug carriers, micro/nano robots and cell/tissue scaffolds. Finally, the current challenges and future perspective of integrated neurobiology research platform are discussed.

## Introduction

Neurological diseases have become one of the leading causes of disability and death in the past decade (Feigin et al., 2020). Based on neuronal synaptic activity and supported by multiple neuroglia cells, the nervous system is highly complex and urgently needs tools in a highly controllable manner to facilitate research in the field. Although the physiological structure and function of the central nervous system (CNS) have been well studied, problems still exist in drug delivery development for CNS diseases (Gribkoff and Kaczmarek, 2017). Fabrication strategies to set up advanced micro/nano-scale devices are still challenging, which hampers the investigation of new medicine.

**FIGURE 1:**
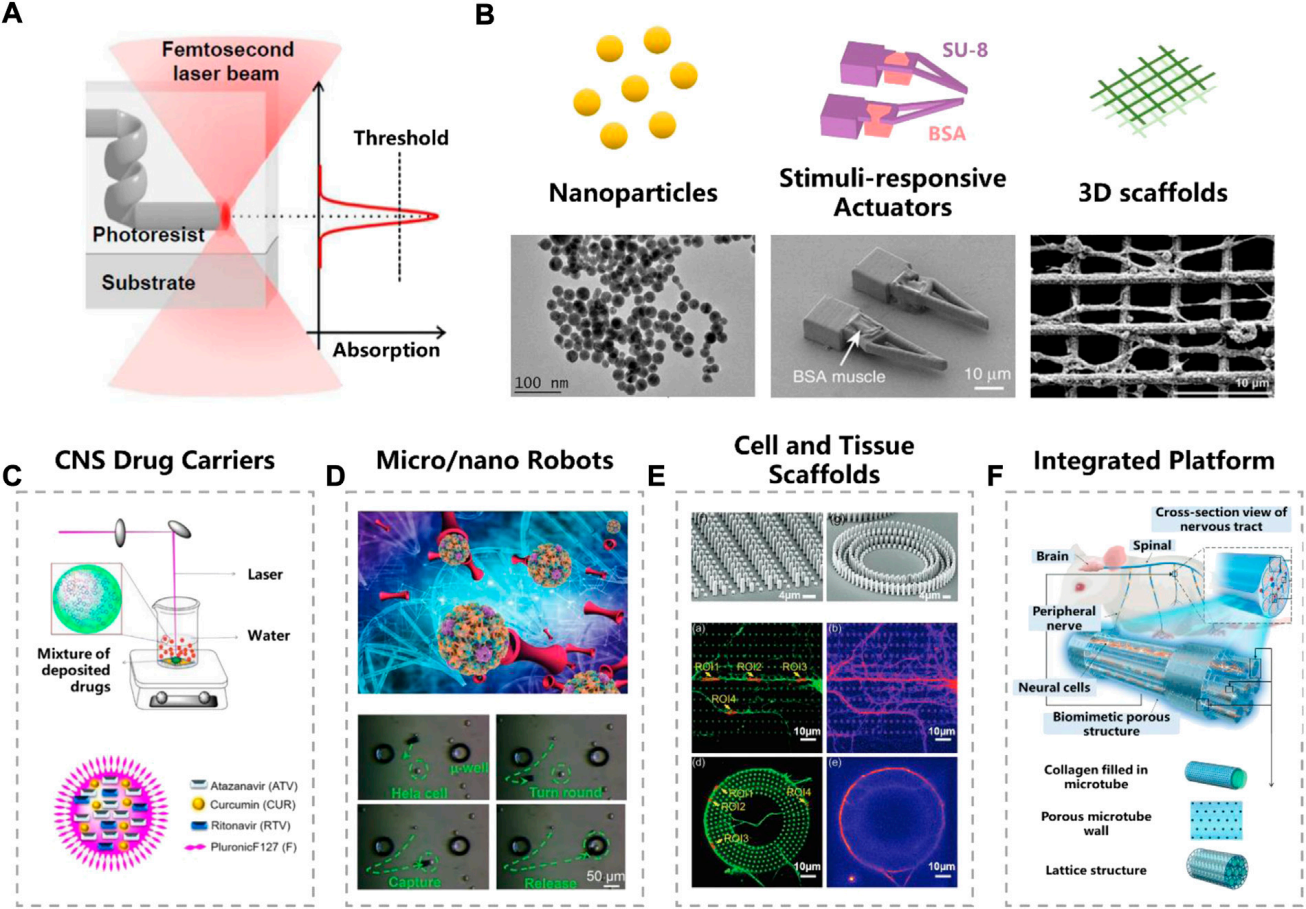
Femtosecond laser fabrication in neurobiological research. **(A)** Femtosecond laser nonlinear absorption mechanism. Reproduced from (Harinarayana and Shin, 2021) with permission of Elsevier. **(B)** Typical micro/nano structures fabricated by femtosecond laser. Reproduced from (Bailly et al., 2019) (Ma et al., 2020) with permission of Springer Nature. Reproduced from (Agrawal et al., 2021) with permission of Elsevier. **(C–E)** Functional units fabricated by femtosecond laser. **(C)** CNS drug carriers. Reproduced from (Singh et al., 2020) with permission of Elsevier. **(D)** Micro/nano robots. Reproduced from (Yang et al., 2019) with permission of Wiley-VCH. **(E)** Cell and tissue scaffolds. Reproduced from (Fan et al., 2021) with permission of Wiley-VCH. **(F)** Integrated platform. Reproduced from (Wei et al., 2022) with permission of Royal Society of Chemistry.

Compared to traditional micro/nano fabrication techniques, femtosecond laser processing is regarded as a promising candidate for biological applications (Zhang et al., 2010), which possesses the merits of both nanoscale feature sizes and 3D architecting capability. Extremely high-power density of the focused laser spot makes the materials absorb these photons in a nonlinear manner (i.e., two- or multi-photon absorption), producing 3D structures with fine resolution beyond the optical diffraction limit (Figure 1A). When adjusting the wavelength, pulse energy and repetition frequency of laser source, various kinds of materials can be printed, such as polymer (Kawata et al., 2001), hydrogel (Ma et al., 2020), metal (Ma et al., 2018), semiconductor and sapphire (Li et al., 2016). The 3D processing capability also makes the fabrication technique a powerful tool to integrate functional structures on a non-planar surface or inside biological tissues.

Owing to the high-precision nature, femtosecond laser fabrication fits well with the high complexity of the nervous system, which offers great advantages for neurobiological applications. The strength of its 3D processing flexibility allows femtosecond laser fabrication to create well-used *in vivo* and *in vitro* tools for neurobiology. In this mini review, we summarize the contributions of femtosecond laser fabrication to the field of neurobiology and neurological diseases. Typical structures used in neuronal science are sorted out from nano, submicron to micron scale, including nanoparticles, micro/nano-size responsive actuators, and macroscopic 3D scaffolds. Then, functional unit composed of these delicate structures in neurobiological scenarios are presented, such as CNS drug carriers, micro/nano robots and cell/tissue scaffolds. Finally, the current challenges and future perspective of integrated neurobiology research platform are discussed.

## Micro/nano structures fabricated with femtosecond laser

The basic concept of femtosecond laser fabrication, including working principle and fabrication strategies, have been comprehensively reviewed by many other researchers (Zhang et al., 2010; Harinarayana and Shin, 2021). Represented by laser ablation and two-photon polymerization (TPP), femtosecond laser is widely used in the fabricating biological microstructures. In this chapter, we introduce the typical micro/nano structures used in neural science research, which are fabricated by femtosecond laser, including nanoparticles, micro/nano actuators and complex stereoscopic scaffolds (Figure 1B).

Femtosecond laser with high power density can ablate the solid material into nanoscale clusters. When put the target materials in a liquid environment such as deionized water, a nanoparticle colloidal solution can be obtained (Aa et al., 2022). Compared to conventional chemical synthesis methods, laser ablation avoids using toxic chemical additives, such as reductant and surfactant, so that the final product has a higher purity. Braguer et al. prepared dextran-modified gold nanoparticles (AuNP), which can easily enter glioblastoma cells without causing toxic effect (Correard et al., 2014; Bailly et al., 2019). By introducing some nerve agents into the liquid, single and multi-drug hybrid nanoparticles can be achieved. Nanoparticles, such as gold (Bailly et al., 2019), silicon (Petriev et al., 2019), titanium nitride (Zelepukin et al., 2021; Aa et al., 2022), and boron (Pastukhov et al., 2022), are often chosen as nano drug carriers or photothermal therapy agents for neurological diseases.

Stimuli-responsive actuators, usually made of two or more kinds of materials (Han et al., 2018). However, the integration of distinct materials at nano/micro-scale is extremely challenging for traditional fabrication techniques. With the development of femtosecond laser fabrication technology, arbitrary 3D micro-actuators can be made (Xin et al., 2019; Yang et al., 2019; Ma et al., 2020; Wang et al., 2021). Ma and their colleagues have developed a pH-responsive microgripper, which integrates the rigid SU-8 skeleton and soft driving material (bovine serum albumin, BSA) together. In addition to fabricating on a planar substrate, a force-sensing microgripper was integrated on the tip of an optical fiber (Power et al., 2018).

TPP is also applied to print 3D cell culturing scaffolds. Since the long-time interaction between scaffolds and cells, material selection is one of the major issues in fabrication. Hydrogel (Enrico et al., 2022), protein (Huang et al., 2020), acrylate-based materials (Nichol et al., 2010) and hybrid materials, have been used in building scaffolds. Hydrogels have the excellent biocompatibility and extracellular matrix (ECM) similarity (Song et al., 2020), such as a BSA scaffold was successfully made by Paul’s group studying the stem cell migration (Su et al., 2012). Considering that hybrid inorganic-organic materials can provide better controllability compared to natural materials, Franziska et al. synthesized a two-component scaffold for 3D cell culture (Klein et al., 2011). All of these scaffolds fabricated by TPP have been deeply involved in neuronal cell culture, tissue engineering, cancer metastasis and other fields. Advanced processing technology represented by TPP will promote the investigation of more potent micro tools, which may greatly facilitate the neurological research.

## Functional unit for neurobiological research

### Central nervous system drug carriers

Most drugs can hardly reach the CNS due to the presence of the biological barrier, such as blood-brain barrier (BBB) and blood–cerebrospinal fluid (CSF) barrier, making the development of drugs for the CNS an extreme challenge. Extensive efforts are made to find proper drug carriers, which can self-directed bypass or cross biological barriers. It is reported that nanoparticles smaller than 100 nm are more likely to cross the BBB (Nance et al., 2022). Eszter et al. reduced the particle size down to submicron scale (50 nm) by laser ablation strategy, and applied them to transnasal drug delivery (Nagy et al., 2021). Furthermore, Ajay et al. reduced the particle size to 20–25 nm, and successfully carried three drugs for HIV-1 (Figure 1C) (Singh et al., 2020). Experiment validated that these nanoparticles showed high permeability in an *in vitro* BBB model. No toxic chemical induced, as well as the tiny size of carriers, make laser ablation an effective way to make CNS drug carriers.

### Micro/nano robots

The excellent maneuverability of micro/nano robots (MNRs) helps to deliver cargoes to target regions precisely and safely. Actuation can be triggered by various stimuli, such as *in vivo* endogenous dynamics (chemical or enzyme) or *in virto* external energy (magnetism, light, pH, microforce, heat, etc.). Robots driven by magnetic field are popular due to the bio-friendly nature (Zhang et al., 2021). Usually, the microrobot was firstly fabricated by TPP strategy, and then followed by depositing magnetic materials (such as Ni/Ti) on the surface of photoresist structures (Li et al., 2018). Complex 3D-shaped micro-robot, such as burr-like porous spherical structure and scaffold-like structures, can be achieved with fine configuration (Jeon et al., 2019). Jiaru et al. modulated the laser vortices and recently made hollow magnetic tubular micromotor and conical microhelices, which significantly improved the swimming ability (Figure 1D) (Xin et al., 2019; Yang et al., 2019). MNRs driven by other external stimuli can also fabricated trough femtosecond laser. Dong and colleagues have developed a pH-responsive microgripper based on a femtosecond Bessel beam that enables rapid capture of neural stem cells (Li et al., 2020). Furthermore, they combined pH response with magnetic propulsion to develop shape-morphing microfish for local cancer treatment (Xin et al., 2021). Many *in vitro* studies and animal experiments have been performed to verify the capability of micro/nano robot for cellular therapy and intra-biofluid regulation (Wei et al., 2020; Wu et al., 2020).

Cellular therapy for CNS diseases is a promising approach that has made extensive progress in recent years for example in the treatment of degenerative neurological diseases (Lindvall et al., 2004). MNRs can achieve localized and controlled cell delivery. Recently, Sun et al. designed a magnetic microrobot with a burr-like porous spherical structure using TPP to achieve cellular release in mice (Li et al., 2018). Mei et al. presented some biodegradable hydrogel micro-robots integrated with an electrode (Dong et al., 2020), so that the cargo delivery and electrical stimulation can be achieved simultaneously for localized neuronal differentiation. However, efforts are still needed to empower these micro/nano robots to better cross the BBB and can be precisely directed to the CNS.

### Cell and tissue scaffolds

Cell culture is an essential step for *in vitro* neurobiology research, however, traditional 2D culture are not quite efficient to mimic *in vivo* physiological environments, encountering excessive cell migration, mechanical mismatch of soft tissue and hard containers, and inappropriate extracellular matrix environment. Different from 2D culturing, 3D scaffold greatly helps improving the spatial ECM, which are widely used in culturing neuronal axons and dendrites with stereoscopic distribution. Highly ordered nanogrids fabricated by femtosecond laser were presented by Agrawal et al. to enhance the directional growth of neuronal cells (Agrawal et al., 2021). Fan et al. used TPP to make micropillar scaffolds for guiding the pattern growth of neuronal axons and dendrites (Figure 1E) (Fan et al., 2021). Restoration and repair of disrupt neuronal are expected under the guidance of these micro/nano structures. In order to study the human nervous system in depth, there is a growing interest in preparation of novel integrated research platform. For example, a bionic cell culture platform inspired by the peripheral nerve bundles have been successfully made for guiding the growth of neural cell (Figure 1F) (Wei et al., 2022). Although these studies are still in their infancy, we believe that they may help to open up the possibility for future life-like computing and artificial intelligence.

## Conclusion and outlook

In conclusion, recent advancement of femtosecond laser fabrication in neurobiological research are briefly summarized in this review. Femtosecond laser fabrication, with the merit of fine resolution and arbitrary 3D constructions, may become a handy tool to promote the development of basic neuronal science. Various 3D micro/nano structures were carried out and adopted in a wide range of applications in the field of neurobiology. Up to now, constant efforts are still needed to investigate high-throughput and large-scale fabrication strategy, including system automation, multi-material fabrication, multi-functional components integration. In addition, photoresists with better biocompatibility also need to be developed, so that less side-effect can be induced in CNS *in vivo*. Besides, 3D micro platform, such as 3D microfluidic systems, are expected to enable high-throughput neuronal culture and drug screening *in virto*. Integrated platform with multi-functional groups may provide opportunities for future CNS drug screening and neuron regeneration studies. We believe that neurological diseases will encounter more adaptive and effective treatment, which serve the interests of people all over the world.
